# Chronic fatigue syndrome and an illness-focused approach to care: controversy, morality and paradox

**DOI:** 10.1136/medhum-2018-011598

**Published:** 2019-06-18

**Authors:** Michael Sharpe, Monica Greco

**Affiliations:** 1 Psychiatry, University of Oxford, Oxford, UK; 2 Sociology, Goldsmiths College, London, UK

**Keywords:** fatigue, chronic fatigue syndrome, mind-body, philosophy, integrated care

## Abstract

Contemporary medicine distinguishes between illness and disease. Illness refers to a person’s subjective experience of symptoms; disease refers to objective bodily pathology. For many illnesses, medicine has made great progress in finding and treating associated disease. However, not all illnesses are successfully relieved by treating the disease. In some such cases, the patient’s suffering can only be reduced by treatment that is focused on the illness itself. Chronic disabling fatigue is a common symptom of illness, for which disease-focused treatment is often not effective, but for which illness-focused treatments (psychological or behavioural) often are. In this article, we explore a controversy surrounding illness-focused treatments for fatigue. We do this by contrasting their acceptance by people whose fatigue is associated with a disease (using the example of cancer-related fatigue) with their controversial rejection by some people whose fatigue is not associated with an established disease (chronic fatigue syndrome or CFS, sometimes called ME (myalgic encephalomyelitis)). In order to understand this difference in acceptability we consider the differing moral connotations of illness and disease and then go on to examine the limitations of the concepts of illness and disease themselves. We conclude that a general acceptance of illness-focused treatments by all who might benefit from them will require a major long-term change in thinking about illness, but that improvements to the care of individual patients can be made today.

## Introduction

Contemporary medicine makes an important distinction between ‘illness’, which is defined by the patient’s subjective experience of symptoms and disability, and ‘disease’, which is defined by objectively observable bodily pathology.1 Medicine typically concentrates its efforts on seeking and treating disease. This focus on disease has the huge advantage of providing a target for disease-focused treatment, but the potential disadvantage of neglecting the patient’s experience of illness. This neglect of illness is particularly problematic when the focus on disease alone does not relieve the patient’s suffering.

The symptom of fatigue is a common and disabling component of many illnesses and one that is often beyond the reach of disease-focused medicine.2 It occurs as part of the illness associated with cancer and often persists after successful cancer treatment. It also occurs as part of the illness of patients with a diagnosis of chronic fatigue syndrome (CFS) (sometimes referred to as myalgic encephalomyelitis or ME, which some consider to be the same illness as CFS and others do not).3 The fatigue in both these cases has been found in research studies to be lessened by illness-focused rehabilitative treatments that include a talking treatment called cognitive–behavioural therapy (CBT) and a behavioural treatment called graded exercise therapy (GET).

However, while these treatments are typically welcomed by patients with cancer, some patients with CFS reject them. These individual rejections have coalesced into vigorous campaigns against the whole idea behind these treatments and consequently a very public and fraught controversy about their validity and place in care.4

In this article, we seek to better understand this controversy. In the first half of the paper, we use a perspective informed by the concepts of illness and disease to explore the moral connotations of this distinction, and in the second half, we examine the limitations of the concepts of illness and disease themselves.

We conclude that a paradox arising from the current concepts of ‘illness’ and ‘disease’ is at least partially responsible for the controversy in the treatment of CFS and make proposals about how it might be addressed.

## Illness, disease and morality: a first-order perspective

If we travelled back to the 18th century, we would find that physicians were treating people for a variety of illnesses, but without any real understanding of the associated disease. Medical practice began to change when observable bodily abnormalities were found to be associated with some forms of illness. The process began in earnest when the dissection of human bodies became more systematic, allowing physicians to associate previously poorly understood illnesses with objectively observable disease pathology. Over time the ability of doctors to find disease inside the body has been greatly enhanced by new technologies such X-ray, measurement of blood chemistry, and latterly by modern imaging and molecular diagnostic techniques. As a result, medicine’s ability to ‘explain’ illness in terms of disease has increased exponentially. Disease-focused medicine has, as a result of this progress, provided effective treatments for many illnesses by creating disease-focused treatments.

Disease-focused medicine has however been less successful in relieving the symptoms of some other illnesses. The experience of chronic disabling fatigue, which is a common component of the illness experience, is such an example. Fatigue is part of the illness associated with the disease we call cancer and often persists despite successful treatment of the disease.5 Fatigue is also part of the illness we refer to as CFS for which, as yet, no generally accepted disease has been identified to treat.6 In both cases, a disease-focused approach to medical care is ineffective in relieving the patient’s illness.

This shortcoming of disease-focused care can be addressed by focusing treatment not on the disease, but on the illness itself. Such illness-focused treatments have been found in research studies to be helpful in relieving both cancer-related fatigue and CFS-related fatigue. They are rehabilitative approaches that involve carefully managed increases in activity and include the talking treatment called CBT and the behavioural treatment called GET.7

While fatigue has been found to be relieved to a similar degree by these treatments in both patients with cancer and patients with CFS, the reception to them has differed markedly: CBT and GET for fatigue are generally accepted by patients with cancer, but sometimes strongly rejected by patients with CFS. In the case of CFS, this rejection has coalesced into active campaigns against their provision. The result is a controversy about the very place of an illness-focused approach to care. 8

We suggest that to better understand this rejection and the resulting controversy, we need first to examine the moral and social connotations of different kinds of illness. We note that patients with illnesses that are associated with a disease, such as cancer, usually benefit from unqualified acceptance of their symptoms and associated disability. In this case, the presence of a disease ensures that the illness is considered ‘genuine’; a moral validation allows patients to claim the benefits of the sick role, including sympathy and exemption from duties, as well as permission to access publicly funded healthcare and other financial benefits.9 Consequently, patients are able to see the application of psychological and behavioural treatments for their fatigue as a benefit.

By contrast, we note that patients whose illnesses have *not* so far been found to be associated with generally accepted bodily disease (such as those with a diagnosis of CFS) find themselves in a much more morally uncertain position. They face the possibility that their experience of illness will be rejected as ‘not real’, with all the implications for acceptance, care and financial support that such a judgement implies.10

The potential consequence of this difference in moral meaning is that for patients with ‘illness-without-disease’ such as CFS, psychological and behavioural therapies for fatigue may not be seen as beneficial. Rather they may be seen as a threat that risks further undermining the moral status of their illness, suggesting that it is ultimately psychological in nature, or even ‘all in the mind’. To the extent that this is the case, it is therefore perhaps not surprising that some of those suffering from CFS reject illness-focused treatments.

In summary, a diagnosis of disease is not only a medical matter; it is also a personal and social matter, providing moral validation of the illness.11 The absence of a disease diagnosis can make an illness appear morally ambiguous. We propose that these different moral connotations and their social consequences may at least partially explain the differing responses to illness-focused treatments. Their rejection by some patients with CFS can consequently be seen not as irrational, but as entirely understandable. In the next part of the paper, we consider the historical and philosophical sources of these moral assumptions, and ask whether and how it might be possible to overcome them.

## Illness, disease and paradox: a second-order perspective

The distinction between disease and illness that we have described is typically used to mark the difference between two different kinds of reality: the ‘objective’ reality of observable bodily pathology, on the one hand; and the ‘subjective’ reality of the felt experience of symptoms and disability on the other. This distinction between realities that are regarded as either ‘objective’ or as ‘subjective’ lies at the core of modern thought, so much so that it is extremely difficult to think at all without it, or beyond it. In medicine, acknowledging the subjective dimension of illness may be regarded as a welcome corrective to the perceived reductionism of the biomedical model, allowing for a more comprehensive and patient-centred conception of the scope of medical care. Yet the dualist logic on which the illness/disease distinction is premised leaves some patients in a difficult and potentially paradoxical predicament. As we shall see, this is because the two concepts (as they are typically used) are not symmetrical, but rather set in a hierarchical relationship.

The logic that informs the illness/disease distinction corresponds to what the philosopher and mathematician Alfred North Whitehead called the ‘bifurcation of nature’.12 Whitehead used this expression to describe a set of assumptions that became established with the emergence and then triumph of modern science, from the 17th century onwards. Physics was the first of the modern sciences, and the conceptual system that it instituted coloured subsequent developments in knowledge, including those in medicine. Founded on experimental practice and on the systematic application of mathematics for the description of relations observable in nature, modern (classical) physics inaugurated ‘scientific materialism’: the view that nature (or reality) consists fundamentally of matter, localisable in space and time, whose properties can be expressed in mathematical and qualitatively neutral terms.

This scientific materialism proved to be formidably efficient for organising research and for subsequent technological development. At the same time, however, it produced an understanding of nature that stood in sharp contrast with ordinary perception. This resulted in a dilemma as to how these two divergent realities, one known only through the mediation of experimental apparatus and mathematical abstractions and the other immediate and sensuous, could be reconciled. ‘The history of thought in the eighteenth and nineteenth centuries’, writes Whitehead, ‘is governed by the fact that the world had got hold of a general idea [scientific materialism] which it could neither live with, nor live without’.13

The concept of a ‘mind’ that is distinct and separate from physical nature has been proposed by philosophers from Descartes to Kant and has become part of modern common sense. It represents a solution to the dilemma generated by the rise of scientific materialism and bridges the gap between reality as described (ultimately) by physics and reality as we immediately experience it in perception. The character of this dualist solution is spelled out most clearly in John Locke’s theory of ‘primary’ and ‘secondary’ qualities of matter.14 The mind, so the theory goes, is prompted into a reaction by the physical and causal properties of matter (‘primary qualities’), but also adds something of its own to produce the qualitative effects—all the sensory impressions, and accompanying judgements of meaning and value—that we experience in perception. These ‘secondary qualities’ (or psychic additions) exist only in the mind rather than in nature as such: *they are therefore of the order of appearance rather than of ultimate reality*.

‘Thus’, writes Whitehead, ‘nature gets credit which should in truth be reserved for ourselves: the rose for its scent: the nightingale for his song: and the sun for his radiance. The poets are entirely mistaken. They should address their lyrics to themselves, and should turn them into odes of self-congratulation on the excellency of the human mind’.15

As can be seen in this quotation, the logic of the bifurcation of nature, when followed to its conclusion, yields arguably absurd propositions. The notion that even a concrete experience (like an illness) can be regarded as being ‘all in the mind’, and therefore ‘not quite real’, can be seen to be deeply rooted in the epistemic compromise that arose in response to the development of modern science, based on the precedent set by physics. While this compromise acknowledges the reality of perceptual experience, it defines it as merely ‘subjective’, secondary or even epiphenomenal. At the same time, it defines ultimate reality as ‘objective’, or as that which exists independently of any subject (or perceiver). A subjective reality can therefore be said to be *truly* real only if it corresponds to objective findings.

The concepts of ‘illness’ and ‘disease’, as they are currently used in medicine, are an expression of this compromise. The realities they refer to are not symmetrical but hierarchical: (subjective) illness is understood to be the *effect* of (objective) disease; the reality of the former must be validated by the material reality of the latter in order to be taken seriously.16 The bifurcation of nature also institutes a fundamental separation between objective ‘facts’ and subjective ‘values’, which extends to a distinction between blind material causality and morally meaningful agency. It is thanks to this separation that, in modernity, the concept of disease—understood as a natural fact—became ‘divorced from the metaphysics of evil, to which it had been related for centuries’.17 In the event of an illness, the presence of associated disease consequently ‘explains’ the illness in terms of blind material causality, and pre-empts any questions as to its moral meaning or value; such questions appear irrelevant, or at least of secondary importance.

The bifurcation of nature, writes Whitehead, ‘has held its own as the guiding principle of scientific studies ever since [the 17^th^ century]. … Every university in the world organises itself in accordance with it’.18 We must stress that it is not only universities, but virtually all modern institutions—legal, educational, economic and medical—that function on the basis of this principle, relying on the difference between ‘objective’ and ‘subjective’ realities for their everyday operations. These operations include gatekeeping decisions that determine access to socially provided facilities and resources, as well as the excusability or otherwise of behaviours that contradict normative expectations. The fact that the distinction between subjective and objective realities is so fundamental to these everyday social functions makes it difficult to challenge scientific materialism in practice—despite the fact that its limitations, especially in the field of medicine, have been theoretically acknowledged for decades.19

The bifurcation of nature therefore produces a paradox whereby the concrete reality we experience can be dismissed as merely delusion, unless it corresponds with objectively known reality identified only through the mediation of technical apparatus. Illnesses that do not correspond to an identified disease pathology are a prime example of this paradox: how can an illness be patently real, sometimes severely so, when doctors can find no good objective evidence of bodily disease to account for it? How can both of the propositions—‘this is a real illness’/‘this is not a disease’—be simultaneously valid and true? Within the logic dictated by the bifurcation of nature they strictly cannot be, because the subjective reality of illness supposedly derives, as we have seen, from the objective reality of disease. This paradox can therefore only be escaped by querying or denying one of its terms.

On one hand, there will be those who seek to escape the paradox by denying the first proposition, casting doubt on whether the person who reports an illness-without-disease is actually ill at all. They may go further to suggest that the person’s reported experience is merely imaginary, deceptive or even the deserved consequence of a personal fault.

On the other hand, there will be those who seek to escape the paradox by denying the second proposition, insisting that the illness is associated with a disease and that the lack of evidence for this is only provisional. They may argue that given enough time and resources, the disease will surely be found (a view associated with preference for the label ME rather than CFS for the illness). The modernist narrative of progress and discovery in which medicine itself is strongly invested reinforces this line of argument.

However, while both ways out of the paradox of illness-without-disease have their advocates, neither fully convinces in the light of evidence for *both* the reality of the illness *and* the absence of conventionally defined disease. Consequently, the paradox is felt, but not resolved.

Is there therefore a better way to resolve the paradox? Following Whitehead, we propose an alternative approach which seeks to examine and revise the concepts of ‘illness’ and ‘disease’ themselves. Instead of thinking of illness and disease in terms of the hierarchical difference between subjective (or mental) and objective (or physical) realities, we propose that we could think of them in terms of different degrees and forms of *abstraction* from the totality of what is real. ‘Abstraction’ is a term Whitehead uses to refer to the activity of abstracting, selecting or bringing something into focus. Abstraction in this sense is a *process* that all organisms engage in as an aspect of their relationship with their environment: our digestive system abstracts ‘food’ from the substances we ingest, for example, while discarding the rest as irrelevant ‘background’. Similarly discrete thoughts are abstracted from an otherwise undifferentiated stream of consciousness, in connection with the demands or features of specific situations.20

The experience of ‘illness’, from this perspective, is a reality selected for perception by an organism as distinct from its normal existence in a negative way, and consequently demanding attention and rectification. This concept of illness points to the perceptual synthesis (or abstraction), made by an organism, of a complex of factors that comprise its situation at a given time, selected for their relevance to the quality of its existence.

‘Disease’, by contrast, involves a higher order of abstraction: its reality is observable only through the coordinated practices and concepts of biomedicine. The reality described as ‘disease’, in other words, is the product of a highly selective focus, both enabled and inherently limited by the apparatus (conceptual and technical) through which it is mediated. We can think of the concept of disease as a highly purified distillate, extracting relevance from the aggregated data of many organisms, with the primary aim of understanding and intervening on the causes of death.21

The abstraction achieved by the concept of disease is hugely powerful: it is the medical equivalent of powerful equations in physics, enabling equally effective ways of acting in the world. From a sociological perspective, the concept of disease (observed or hypothetical) is also the ‘lens’ through which the medical system observes the world and makes decisions as to which realities belong within its remit, and are therefore *really* medical, and which do not, and are therefore only *apparently* medical. As already noted, the privilege accorded to ‘disease’ for medical explanation and treatment is frequently enabling. However, it can also become an instance of what Whitehead called the ‘Fallacy of Misplaced Concreteness’, the mistaking of an abstraction for the concrete reality of what matters in a given situation.22

We have offered a redescription of the concepts of 'illness' and 'disease' that avoids casting them respectively as a subjective (epiphenomenal) reality of the mind and an objective (causal) reality of the body. This redescription allows us to apprecia te that illness and disease are both equally concrete realities, and both equally 'organic' (i.e. of the organism). The relationship between illness and disease is neither necessarily symmetrical, nor hiearchical; rather they index different realities, which sometimes correlate and sometimes do not. We propose this redescription, not as mere wordplay, but as a way of retaining what is useful about the illness/disease distinction, whilst avoiding the pitfalls of the bifurcation of nature.

## Resolving the paradox

As things stand today, the differing moral connotations of illness-with-disease and illness-without-disease remain difficult to overcome in practice. We propose that this is not through a failure of goodwill on anybody’s part but simply because they are so deeply embedded in the semantic fabric through which we make sense of the world. We can therefore no more easily shed these connotations than we can shed the distinction we routinely make between subjective and objective, and between mental and material realities. Versions of this distinction, as we have seen, are fundamental to many social practices and inform the working of virtually all modern institutions. While this does not mean that they are necessary in any absolute sense, it does mean that overcoming them presents a formidable challenge.

With this in mind, we can gain some perspective on both the value and the limitations of an illness-focused approach to the treatment of illnesses-without-disease. On the one hand, in so far as treatments such as CBT and GET relieve the symptom of fatigue, they offer a useful improvement of a condition for which there was previously no treatment at all. But on the other hand, even the idea of such illness-focused treatments may be rendered unacceptable by the paradox of illness-without-disease, whereby the reality of illness can be queried and dismissed as ‘all in the mind’.

Beyond the offer of practical help for the person with fatigue is the wider cultural, social and institutional context which adheres to the bifurcated, reductionist models of what it means for an illness to be ‘real’. Consequently, the meaning and effectiveness of illness-focused treatments depend on these critical contextual factor as much as on their efficacy in research studies. To the extent that patients hope for an improvement of their symptoms and for a future in which their illness will be treated with the same dignity and recognition as any illness-with-disease, illness-focused treatments may appear disappointingly insufficient at best and positively threatening at worst. As we have argued, it is therefore understandable that some patients with CFS oppose illness-focused treatment for fear that the illness-without-disease paradox may imply that their illness might then be dismissed as ‘all in the mind’.

Ironically, one potential effect of the controversy about illness-focused treatment is to reinforce the very epistemic assumptions that gave rise to the paradox of illness-without-disease in the first place. By adhering to the logic that imposes a stark alternative between mind and body, and by underscoring the privilege accorded to the concept of bodily disease, the cultural prejudice against illness-without-disease is strengthened. To the epistemic paradox of illness-without-disease, we might then add a second paradox—one whereby the well-intentioned campaigning for better recognition of CFS inadvertently reinforces the social and cultural conditions that made that recognition more difficult in the first place.

Can we escape the trap set by the paradox of illness-without-disease? Our second-order analysis suggests that we acknowledge that all illnesses are real and arise from a mixture of biological, psychological and social factors.23 This new approach assumes that the reality of illness has a complex and indeterminate character, and that the presence of disease does not indicate a more fundamental (or more ‘real’) reality. The focus of medicine on disease is then seen as merely a pragmatic way of simplifying the complexity of illness for the purposes of intervention, valuable where such a simplification is possible and relevant, but also potentially unhelpful or even damaging where it is not. This new approach would therefore fundamentally unsettle the privilege of disease as the arbiter of the validity of illness, and as the model of explanation *par excellence*.

If we wanted to achieve such a change in thinking, we would need to educate and persuade both doctors and the public to overcome their long-standing ‘bifurcated’ habits of thought. In other words, we would need to develop ways of thinking, communicating and making decisions that arguably better reflect the insights and explanatory models of the 21st-century natural sciences, than those of the 17th.24 Over the longer term, if successful, such a change in thinking could give rise to a new ‘social contract of health’. This new contract would no longer be exclusively reliant on the presence or absence of disease as the arbiter of the moral validity of an illness and would not impose a choice between ‘physical’ and ‘mental’ modes of characterisation. It would therefore be better suited to recognising and validating the many illnesses, for which a focus solely on disease is inadequate. However, since our current concept of disease is so fundamental to the performance of important social functions—including validating illness and gatekeeping access to medical care and financial benefits—we must be mindful that such a change would require major societal adjustment and would therefore likely be resisted, unless workable alternative mechanisms for achieving these (or equivalent) functions were also created.

To achieve such a change in the care of individual patients may however be easier and could potentially be achieved much more quickly. 25 To do this, doctors would simply need to be aware of the illness-without-disease paradox that their patients have to manage, to acknowledge it and to address it head on. As a minimum, this requires three steps: The essential first step is to compassionately validate the reality of the patient’s suffering, even in the absence of a demonstrable disease. The second step is to develop the dialogue of the consultation beyond a preoccupation with the presence or absence of disease, towards a consideration of the illness itself. The third step is to explore with the patient, how their illness might be improved and how they might manage the paradox of illness-without-disease in their own life.

## Conclusions

We have inherited a dichotomous and ‘bifurcated’ way of looking at illness. According to this dichotomy, illnesses associated with disease are distinguished from those that are not. Modern medicine is disease-focused and privileges observable evidence of pathology over the experience of illness when determining what constitutes medical reality, with the associated moral connotations of this determination. While hugely successful, this hierarchical perspective leaves patients who have illnesses-without-disease in a paradoxical and potentially harmful predicament. We have argued that this paradox is an important and understandable factor behind the controversy about the illness-focused approach to CFS. We have further argued that, while illness-focused treatments like CBT and GET can ameliorate the symptom of fatigue, their general acceptance requires more than evidence for their efficacy; it also requires that we address the paradoxical predicament of illness-without-disease that patients find themselves in. While at a social level addressing the paradox is likely to necessitate the development of new thinking and institutional processes, at an individual patient level, doctors can address the paradox today by being aware of how it traps their patients and helping them to escape.

## Notes

1. Leon Eisenberg (1977), “Disease and illness. Distinctions between professional and popular ideas of sickness,” *Culture Medicine and Psychiatry* 1: 9–23.

2. Simon Wessely, Matthew Hotopf and Michael Sharpe (1998), *Chronic Fatigue and Its Syndromes* (Oxford: Oxford University Press).

3. Kjetil Gundro Brurberg et al. (2014), “Case Definitions for Chronic Fatigue Syndrome/Myalgic Encephalomyelitis (CFS/ME): A Systematic Review,” *BMJ Open* 4, no. 2: 1–12.

4. Kate Kelland (2018), “Exclusive: Science Journal to Withdraw Chronic Fatigue Review Amid Patient Activist Complaints,” *Reuters*, October 17, 2018, https://uk.reuters.com/article/us-health-chronicfatigue-dispute/exclusive-science-journal-to-withdraw-chronic-fatigue-review-amid-patient-activist-complaints-idUKKCN1MR2PI

5. Petra Servaes, Stans Verhagen and Gijs Bleijenberg (2002), “Fatigue in Cancer Patients during and after Treatment: Prevalence, Correlates and Intervention,” *European Journal of Cancer* 38, no. 1: 27–43.

6. Keiji Fukuda et al. (1994), “The Chronic Fatigue Syndrome: A Comprehensive Approach to Its Definition and Study,” *Annals of Internal Medicine* 121, no. 12 (1994): 953–59.

7. Marieke FM Gielissen, and Gijs Bleijenberg (2009), “Psychosocial Interventions for Reducing Fatigue During Cancer Treatment in Adults,” *Cochrane Database of Systematic Reviews* 110, no. 6: 1–66; Ellen van Weert et al. (2010), “Cancer-Related Fatigue and Rehabilitation: A Randomized Controlled Multicenter Trial Comparing Physical Training Combined with Cognitive-Behavioral Therapy with Physical Training Only and with No Intervention,” *Physical Therapy* 90, no. 10: 1413–25; Lillebeth Larun et al. (2017), “Exercise Therapy for Chronic Fatigue Syndrome,” *Cochrane Database of Systematic Reviews*: 1–138; PD White et al. (2011), “Comparison of Adaptive Pacing Therapy, Cognitive Behaviour Therapy, Graded Exercise Therapy, and Specialist Medical Care for Chronic Fatigue Syndrome (PACE): A Randomised Trial,” *The Lancet* 377, no. 9768: 823–36.

8. Charles Shepherd (2016), “Patient Reaction to the PACE Trial,” *Lancet Psychiatry* 3, no. 2 (February 2016): e7–8, https://doi.org/10.1016/S2215-0366(15)00546-5; Keith J Geraghty and Charlotte Blease (2018), “Myalgic Encephalomyelitis/Chronic Fatigue Syndrome and the Biopsychosocial Model: A Review of Patient Harm and Distress in the Medical Encounter,” *Disability and Rehabilitation* 270, no. 1 (June 2018): 1–10, https://doi.org/10.1080/09638288.2018.1481149

9. Talcott Parsons (1951), *The Social System* (New York: Free Press of Glencoe).

10. Laurence J. Kirmayer (1988), “Mind and Body as Metaphors: Hidden Values in Biomedicine,” in *Biomedicine Examined*, ed. M. Lock and D. Gordon (Dordrecht: Kluwer), 57–92.

11. Annemarie Jutel and Sarah Nettleton (2011), “Towards a Sociology of Diagnosis: Reflections and Opportunities,” *Social Science and Medicine*, 73, no. 6: 793–800.

12. Alfred North Whitehead (1927), *The Concept of Nature* (New York: Prometheus Books, 2004 (1920)), 30.

13. Alfred North Whitehead (1926), *Science and the Modern World* (London, Free Association Books) 64.

14. John Locke (1997), *An Essay Concerning Human Understanding* (London: Penguin Books, 1997 [1690]).

15. Whitehead, *Science and the Modern World*, 68–9.

16. Horacio Fabrega (1990), “The Concept of Somatization as a Cultural and Historical Product of Western Medicine,” *Psychosomatic Medicine* 52, no. 6: 653–72.

17. Michel Foucault (1976), *The Birth of the Clinic: An Archaeology of Medical Perception* (London: Tavistock), 196.

18. Whitehead, *Science and the Modern World*, 69.

19. George L. Engel (1977), “The Need for a New Medical Model: A Challenge for Biomedicine,” *Science* 196: 129–96; Christopher Lawrence and George Weisz (2001) (eds), *Greater than the Parts: Holism in Biomedicine, 1920–* *1950* (Oxford: Oxford University Press, 1998); Laurence Foss (2001), *The End of Modern Medicine: Biomedical Science under a Microscope* (New York: State University of New York Press).

20. Alfred North Whitehead (1927), *Symbolism: Its Meaning and Effect* (New York: Fordham University Press).

21. Michel Foucault (1976) in *The Birth of the Clinic* gives a powerful account of the epistemic centrality of death to the structure of anatomo-clinical medicine.

22. Whitehead, *Science and the Modern World*, 72.

23. Engel, “The Need for a New Medical Model,” 129–36.

24. Ilya Prigogine and Isabelle Stengers (2017), *Order Out of Chaos: Man’s New Dialogue with Nature* (London and New York: Verso Books).

25. Michael Sharpe, “‘Unexplained’ Somatic Symptoms, Functional Syndromes, and Somatization: Do We Need a Paradigm Shift?” *Annals of Internal Medicine* 134, no. 9 (May 2001): 926–30.
